# Diagnostic potential of serum extracellular vesicles expressing prostate-specific membrane antigen in urologic malignancies

**DOI:** 10.1038/s41598-021-94603-9

**Published:** 2021-07-22

**Authors:** Kyojiro Kawakami, Yasunori Fujita, Taku Kato, Kengo Horie, Takuya Koie, Keitaro Umezawa, Hiroki Tsumoto, Yuri Miura, Yasuo Katagiri, Tatsuhiko Miyazaki, Ikuroh Ohsawa, Kosuke Mizutani, Masafumi Ito

**Affiliations:** 1 Research Team for Mechanism of Aging, Tokyo Metropolitan Institute of Gerontology, 35-2 Sakae-cho, Itabashi-ku, Tokyo, 173-0015 Japan; 2 Biological Process of Aging, Tokyo Metropolitan Institute of Gerontology, 35-2 Sakae-cho, Itabashi-ku, Tokyo, 173-0015 Japan; 3 Department of Urology, Asahi University Hospital, 3-23 Hashimotocho, Gifu, Gifu 500-8523 Japan; 4 Department of Urology, Gifu University Graduate School of Medicine, 1-1 Yanagido, Gifu, Gifu 501-1193 Japan; 5 Department of Pathology, Gifu University Hospital, 1-1 Yanagido, Gifu, Gifu 501-1193 Japan

**Keywords:** Prostate, Diagnostic markers, Cancer, Kidney diseases, Diagnostic markers, Translational research, Immunological techniques, Prostate cancer, Renal cancer

## Abstract

We aimed to develop a sandwich ELISA to detect prostate-specific membrane antigen (PSMA) on small extracellular vesicles (EVs) using T-cell immunoglobulin domain and mucin domain-containing protein 4 (Tim4) as a capture molecule for EVs and to evaluate its diagnostic potential in urologic malignancies. First, we optimized the conditions for sandwich ELISA measuring the PSMA level on EVs captured from serum by Tim4 and found that the use of highly-purified EVs released from Tim4 that had captured EVs in serum reduced the background. Second, we confirmed its validity by studying mouse xenograft model for prostate cancer (PC). Lastly, we measured PSMA-EVs in serum of patients with urologic malignancies. The PSMA-EV levels were significantly higher in metastatic PC and castration-resistant PC (CRPC) patients than in therapy-naïve PC patients. In renal cell carcinoma (RCC) patients, PSMA-EVs were elevated in those with metastasis compared with those without metastasis, which may reflect the development of the neovasculature positive for PSMA in tumors. In conclusion, we developed a sandwich ELISA for detection of PSMA-EVs using highly-purified EVs isolated from serum by Tim4. Our results suggest that PSMA-EVs may be useful to diagnose and monitor not only PC but also RCC and possibly other hypervascular solid tumors.

## Introduction

Extracellular vesicles (EVs) are membrane vesicles that are released from cells to the extracellular space, which consist of exosomes (EXs), microparticles (MPs) and apoptotic bodies (ABs)^1^. EXs (50–150 nm in diameter) are generated via the endocytic pathway, MPs (0.1–1 μm) are produced by budding of the plasma membrane and ABs are released from dying cells during program cell death. Since these EV subpopulations in part overlap with each other, EVs are often divided into two categories termed as small (< 200 nm) and large EVs (200 nm^−1^ μm), which sediment at 100,000 × *g* and 10,000 × *g*, respectively^2^.

EVs contain a variety of biological molecules that were present in the cells from which they were derived such as nucleic acids, proteins, and lipids^3^. EVs released from cells not only exist in the microenvironment nearby the host cells but also are present in blood and transferred to distant sites by systemic circulation. For the past decade, it has been demonstrated that EVs are multifunctional vesicles that can exert a variety of biological effects and that EVs in body fluids such as blood and urine can be a promising biomarker for a number of diseases. In the field of cancer research, earlier studies demonstrated that EVs secreted from cancer cells determine the site where metastasis takes place^4^ and that EVs in blood are useful for diagnosis of colon and pancreatic cancer^5,6^ and thereafter numerous reports have been published about their function and potential as a biomarker.

The most commonly used EV isolation method is sequential centrifugation^1^. However, this method is time-consuming and samples prepared from serum contain a substantial amount of serum constituents such as proteins and lipids. Although polymer-based precipitation method is quick, a large quantity of contaminants is precipitated as well. Immunoaffinity-based method such as that using antibody against an exosome surface marker CD9 or CD63 is used for EV isolation, but even with this method contamination is unavoidable. Most of the conventional methods and commercially-available kits for EV isolation work well, when EVs are isolated from cell culture media containing no or a small amount of serum. However, contamination with serum or plasma constituents is inevitable, when EVs are isolated or captured from whole serum or plasma or samples containing them at high concentrations. The contamination is problematic to develop an immunoassay such as ELISA, especially when dealing with targets that are of low abundance in blood such as cancer-derived EVs. The non-specific binding of serum or plasma constituents to the vessels such as microplates and beads as well as to target proteins causes high background and low sensitivity, which is referred to as the “matrix effect”^7^.

Recently, Nakai et al*.* have reported a novel affinity-based method to isolate highly-purified EVs by using T-cell immunoglobulin domain and mucin domain-containing protein 4 (hereinafter referred to as Tim4)^8^. Tim4 is a type I transmembrane protein expressed on phagocytes and immune cells and directly binds to phosphatidylserine (PS) on apoptotic cells. PS, which is usually facing the cytoplasm, gets flipped to the extracellular side of the plasma membrane during apoptosis, by which PS is recognized by Tim4 and other cell surface receptors on phagocytes^9^. The characteristics of the reversed PS are shared by EVs including EXs, MPs, and Abs^10,11^. The newly-developed Tim4-based EV isolation method employed the extracellular region of Tim4 immobilized on magnetic beads to capture EVs. Tim4-binding to PS is Ca^2+^-dependent and intact EVs are readily released by adding chelating agents such as EDTA.

Prostate-specific membrane antigen (PSMA), also known as glutamate carboxypeptidase II, is a type II membrane glycoprotein that is predominantly expressed in prostate and at lower levels in other organs such as brain, kidney, small intestine and salivary gland^12^. In prostate, PSMA is expressed in primary and metastatic prostate cancer (PC) as well as in the epithelium of normal and hyperplastic tissues^13^. In PC patients, PSMA expression was correlated with the stage and Gleason score^14,15^ and with recurrence^16,17^ and was elevated in patients with castration-resistant PC (CRPC) patients^15,18^. On the other hand, it was reported that the endothelial cells in the neovasculature of solid tumors such as renal cell carcinoma (RCC) and breast and colorectal cancer were positive for PSMA, whereas those in the vasculature of normal tissues were negative^19–21^.

In recent years, there has been increasing focus on PSMA-targeted imaging and therapy of PC. PSMA-positron emission tomography/computed tomography (PSMA-PET/CT) has been proven to be effective to diagnose and monitor metastatic PC and CRPC patients and is currently in clinical practice^22^. Since PSMA is also known to be expressed in the neovasculature of hypervascular solid tumors, PSMA-PET/CT has been applied to RCC, demonstrating a potential role in the diagnosis of metastatic RCC patients^23^.

There have been several reports in which the PSMA levels in blood were determined in PC patients. Since PSMA is a membrane-bound protein, it would be possible that PSMA may be shed into circulation^24^, but there appears no convincing evidence for the nature of PSMA in blood. PSMA expressed on the surface of EVs and PSMA released from dying cancer cells may be included in the measured value depending on the method used. Regarding the PSMA-expressing EVs (hereafter referred to as PSMA-EVs), we previously demonstrated their presence in serum of PC patients and elevation in CRPC patients^25^. Since then, there have been a few reports that determined the PSMA-EV levels in PC patients. One group collected EVs by polymer-based precipitation method and detected PSMA by using commercially-available PSMA ELISA kit^26^, while another group measured PSMA-EVs including large EVs by using a commercially-available nano-flow cytometer^27,28^.

In the present study, we aimed to develop a sandwich ELISA to measure PSMA on small EVs in serum. Since our preliminary experiments suggested the difficulty in constructing the system with low background and high sensitivity using conventional methods, we employed Tim4 as a capture molecule for EVs. After optimizing the experimental conditions and confirming the specificity of the PSMA signal, we successfully developed PSMA-EV sandwich ELISA measuring its level on highly-purified EVs isolated from serum by Tim4. We then confirmed the validity of the sandwich ELISA by studying mouse xenograft model for PC and lastly determined the PSMA-EV levels in patients with PC and RCC and evaluated their diagnostic potential.

## Results

### Development of PSMA-EV sandwich ELISA

Since we aimed to determine the PSMA levels in small EVs, serum samples were centrifuged before storage or use at 16,500 × *g* for 30 min to remove large EVs. The particle size distribution of EVs isolated from serum by ultracentrifugation with or without prior centrifugation at 16,500 × *g* is shown in Supplementary Fig. S1. Thus, the term EVs corresponds to small EVs when described for development of sandwich ELISA.

Our preliminary experiments suggested that the development of PSMA-EV sandwich ELISA was difficult when EVs were captured from serum by antibody. We tested all possible combinations of various commercially-available antibodies against CD9 or CD63 and PSMA. However, no combination worked due to high background and low specificity (data not shown). This may be in part because the absolute amount of PSMA-EVs in serum is much less than anticipated. We hypothesized that the use of methods providing higher EV capture efficiency would help detect PSMA-EVs in serum. To achieve this, we employed Tim4 as a capture molecule to increase the yield of EVs from serum.

#### EV capture from serum by Tim4 and anti-CD9 antibody

**Figure 1:**
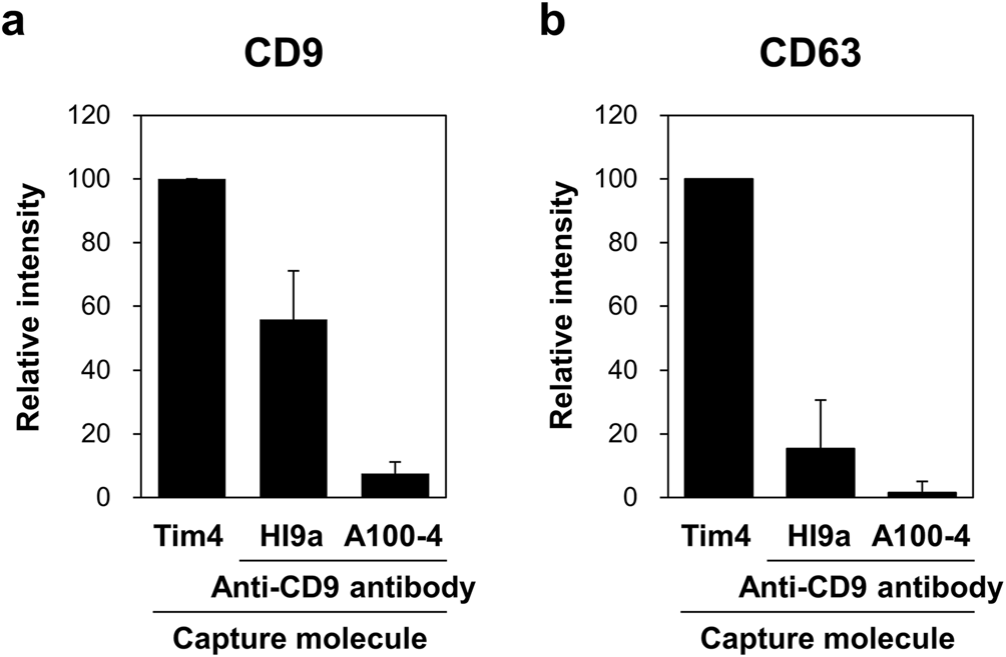
Comparison of the capture efficiency of EVs from serum between Tim4- and anti-CD9 antibody-based methods. Serum of normal donors (n = 6, 50 µL) was applied to a well of 96-well plate coated with Tim4 or anti-CD9 antibody (HI9a or A100-4). After washing, ALP-labeled antibody against an exosomal surface marker CD9 (**a**) or CD63 (**b**) was added to the well, followed by detection of chemiluminescence in the presence of ALP substrate. The signal intensity is expressed as a ratio to that obtained with Tim4. Data are shown by mean ± SD.

We compared the capture efficiency of EVs from serum between Tim4- and anti-CD9 antibody-based methods. Serum was applied to a well of 96-well plate coated with Tim4 or anti-CD9 antibody (HI9a from BioLegend or A100-4 from MBL). After washing, ALP-labeled antibody against an exosomal surface marker CD9 or CD63 was added to the well, followed by detection of chemiluminescence in the presence of ALP substrate. The results showed that the amounts of CD9- and CD63-positive EVs captured by Tim4 was much higher than those captured by anti-CD9 antibodies (Fig. 1a,b, respectively), indicating that Tim4-based method may be more effective than conventional anti-CD9 antibody-based method in capturing EVs from serum.

#### Optimization of sandwich ELISA to detect PSMA on Tim4-captured EVs

In an attempt to develop a sandwich ELISA to detect PSMA on EVs captured by immobilized Tim4, we performed several experiments to determine optimal conditions as described in the following subsections. As a positive control for EVs expressing PSMA, those isolated by sequential centrifugation from culture medium of human androgen-refractory PC C4-2B cells were prepared (hereafter referred to as C4-2B EVs). Increasing doses of C4-2B EVs or serum containing them were applied to a well of 96-well plate coated with Tim4. After washing, ALP-labeled anti-PSMA antibody was added to the well, followed by detection of chemiluminescence in the presence of ALP substrate.

**Figure 2:**
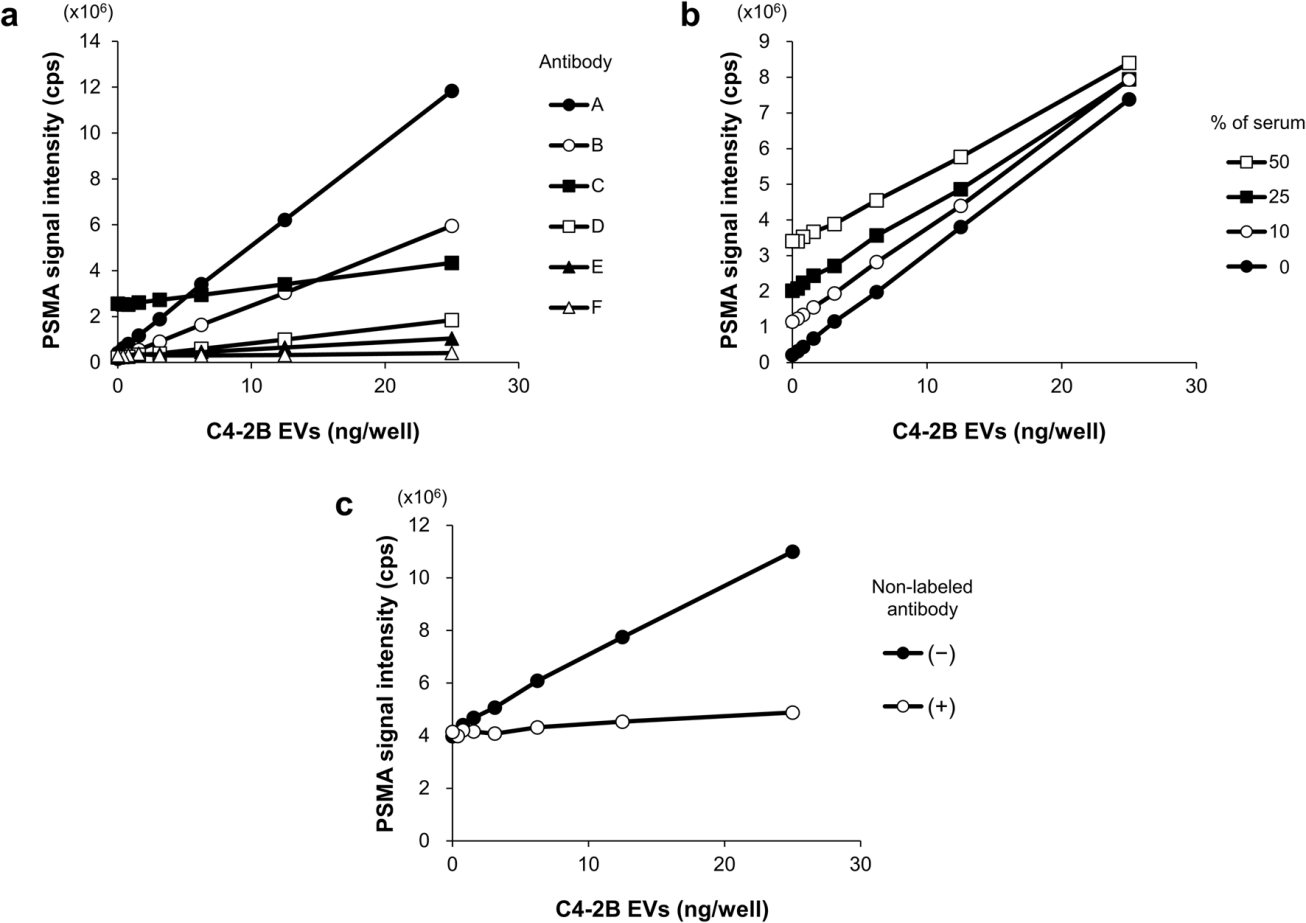
Determination of the optimal conditions for the plate-based PSMA-EV sandwich ELISA. EVs isolated by sequential centrifugation from culture medium of C4-2B cells (C4-2B EVs) were used as a positive control for PSMA-expressing EVs. Samples were applied to a well of 96-well plate coated with Tim4. After washing, ALP-labeled anti-PSMA antibody was added to the well, followed by detection of chemiluminescence in the presence of ALP substrate. (**a**) Antibody selection. Commercially-available anti-PSMA antibodies (A: Miltenyi Biotec, REA408; B: BioLegend, LNI-17; C: MBL, 107-1A4; D: Abcam, GCP-05, E: R&D, sheep-polyclonal; F: Millipore, 3/A12) were labeled with ALP. The PSMA-EV levels in samples containing increasing doses of C4-2B EVs (0, 0.39, 0.78, 1.56, 3.13, 6.25, 12.5, and 25 ng/well in 50 µL) were determined. (**b**) Matrix effect on sandwich ELISA. Measurement of the PSMA-EVs in C4-2B EVs was performed in the presence of increasing concentration of pooled serum from 6 normal donors (0, 10, 25, and 50%). (**c**) Competitive sandwich ELISA. Measurement of the PSMA-EVs in pooled serum containing increasing doses of C4-2B EVs (50 µL) was performed in the presence and absence of an excess amount of non-labeled anti-PSMA antibody (fivefold).

First, we labeled several kinds of commercially-available anti-PSMA antibodies with ALP and examined their compatibility with Tim4. Among them, the REA408 antibody from Miltenyi Biotec (A) was judged to be the best, because of higher sensitivity or the dose-dependent increase in the signal intensity and lower background or the signal in the absence of C4-2B EVs (Fig. 2a). In subsequent studies, therefore, the REA408 antibody was used as a detection antibody for PSMA.

Second, we assessed the specificity of the PSMA signal on EVs by measuring PSMA-EVs in conditioned medium of PSMA knockdown cells. The PSMA-EV levels were lower in the medium of C4-2B cells transfected with PSMA siRNAs than in the medium of those transfected with control siRNAs (Supplementary Fig. S2), indicating that the sandwich ELISA combined with Tim4 and anti-PSMA antibody specifically detects PSMA-expressing EVs.

Third, we studied the matrix effect of serum on the PSMA-EV sandwich ELISA. Using the PSMA-EV sandwich ELISA in which anti-CD9 antibody was used as a capture molecule, a dose-dependent signal was observed for C4-2B EV samples without serum, whereas the presence of serum even at 10% almost completely inhibited the signals (Supplementary Fig. S3). In sharp contrast to this observation, the PSMA-EV sandwich ELISA using Tim4 as a capture molecule showed a dose-dependent signal for C4-2B EVs even at higher serum concentrations up to 50%, although the background signal increased as the serum concentration elevated (Fig. 2b). When detecting PSMA-EVs in serum, hereafter we performed EV capture by Tim4 in the presence of 50% serum to maximize the yield of EVs from serum.

Fourth, we assessed the specificity of the PSMA-EV signal and the nature of the background signal by adding non-labeled anti-PSMA antibody into the reaction. The dose-dependent signal of PSMA-EVs in the presence of 50% serum containing increasing doses of C4-2B EVs was completely inhibited by an excess amount of non-labeled antibody, whereas the background signal was not affected (Fig. 2c). These results indicate that the dose-dependent signal is specific to PSMA-EVs and that the background signal may be due to the matrix effect of serum and ALP activity in serum.

#### EV purification from serum by Tim4 and anti-CD9 antibody

**Figure 3:**
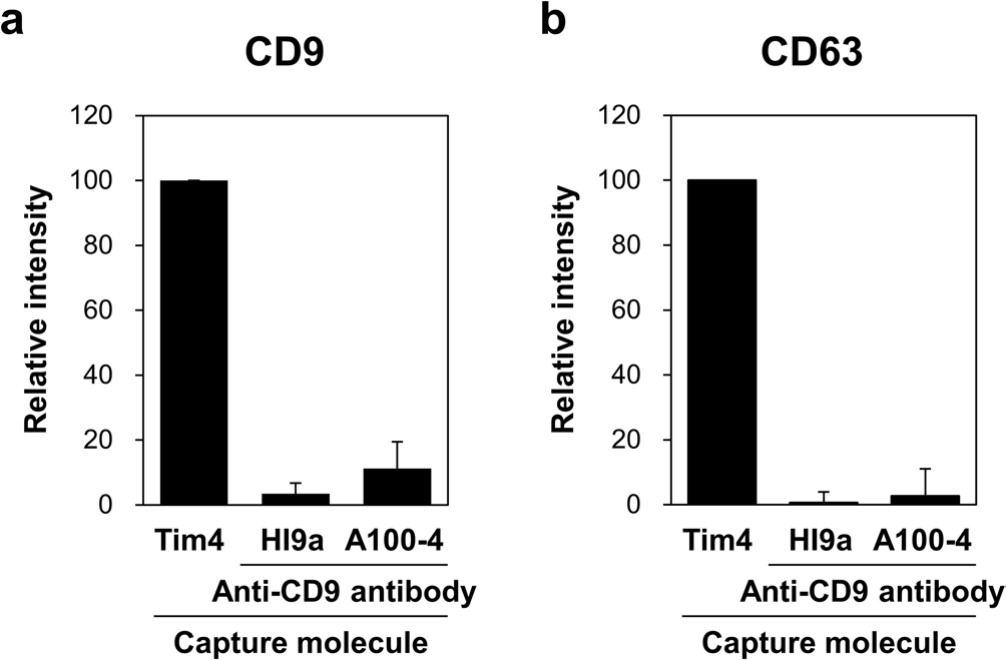
Comparison of the yield of purified EVs from serum between Tim4- and anti-CD9 antibody-based methods. Serum of normal donors (n = 6, 50 µL) was incubated with magnetic beads on which Tim4 or anti-CD9 antibody (HI9a or A100-4) was immobilized. After washing, bound EVs were released from Tim4 and anti-CD9 antibody by adding EDTA and glycine–HCl, respectively. Then, the purified EVs were subjected to bead-based sandwich ELISA for exosomal surface markers CD9 (**a**) and CD63 (**b**). The signal intensity is expressed as a ratio to that obtained with Tim4. Data are shown by mean ± SD.

The results of the competitive sandwich ELISA above suggested the possibility that elimination of matrix components and ALP activity present in serum may reduce the background of the PSMA-EV sandwich ELISA. The physical interaction between Tim4 and PS on EVs is readily disrupted by the addition of a calcium ion chelator EDTA. Similar to proteins, EVs can also be eluted from antibody by lowering pH^29^. We released EVs bound to Tim4 or anti-CD9 antibody that was immobilized on magnetic beads by adding EDTA or glycine–HCl, respectively, obtaining “purified EVs”. The purified EVs were subjected to bead-based sandwich ELISA for CD9 and CD63. Based on the signal intensity of CD9- and CD63-positive EVs (Fig. 3a,b), Tim4-based method isolated at least more than tenfold larger amounts of EVs than anti-CD9 antibody-based methods, showing that Tim4-based method also has an advantage over antibody-based method in terms of the yield of purified EVs.

#### PSMA-EV sandwich ELISA with and without purification of EVs from serum

**Figure 4:**
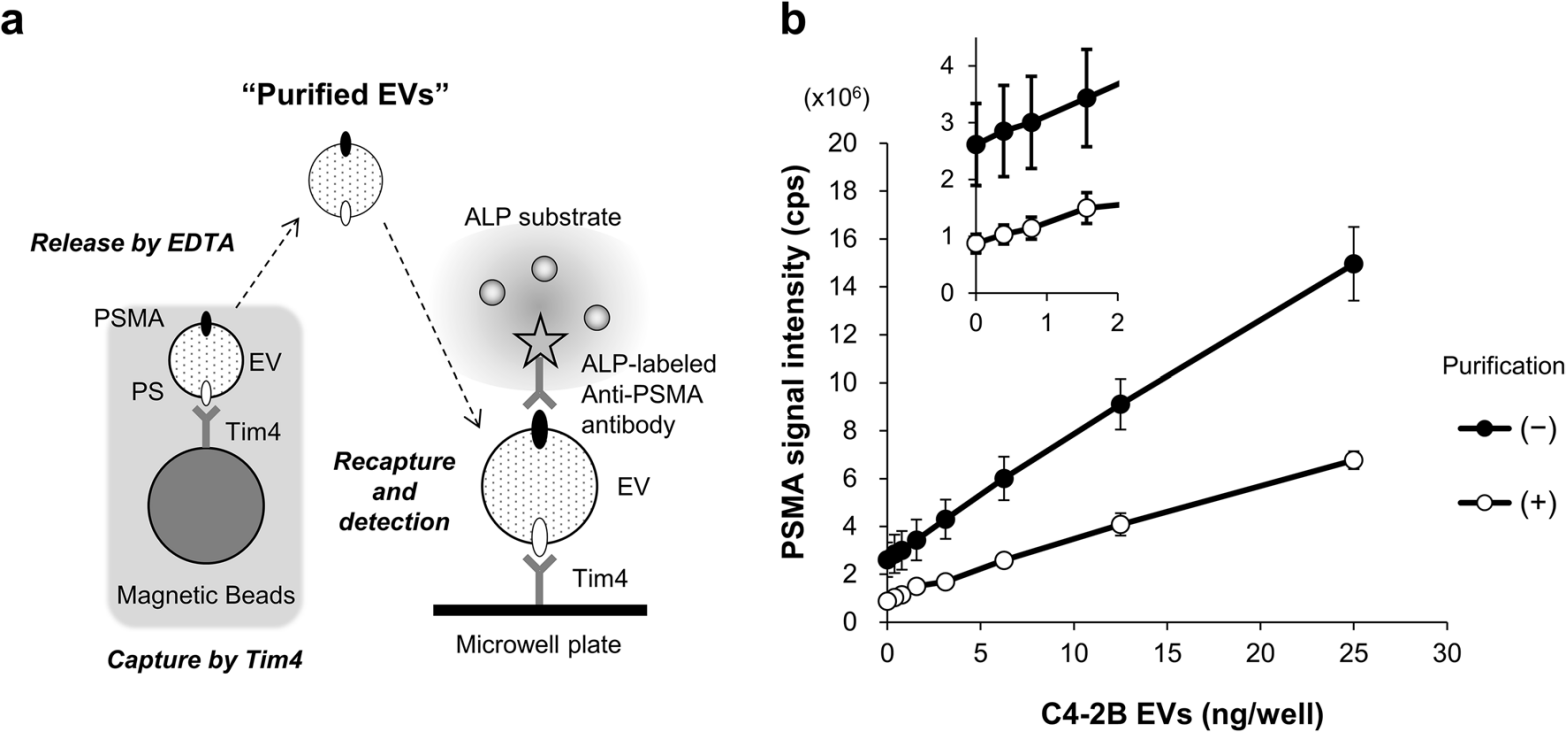
Comparison of the plate-based PSMA-EV sandwich ELISA with and without purification of EVs from serum. (**a**) Schematic diagram of the preparation of “purified EVs” by Tim4 and the measurement of the PSMA levels on EVs recaptured by Tim4 immobilized on the microplate. (**b**) Serum containing increasing doses of C4-2B EVs was prepared for each normal donor (n = 5). The serum containing C4-2B EVs was incubated with Tim4-bound magnetic beads and then EVs were released from Tim4 by adding EDTA. Serum containing increasing doses of C4-2B EVs (50 µL, filled circle) or EVs purified from the same amount of the serum (open circle) were transferred to a well of 96-well plate where they were recaptured by immobilized Tim4, followed by detection of PSMA on EVs. When PSMA-EVs in serum containing C4-2B EVs was measured, EV binding to Tim4 was conducted at its final concentration of 50%. Inset: Enlarged plot at low concentrations. Data are shown by mean ± SD and those for each donor is presented in Supplementary Fig. S4.

We determined the amount of PSMA on purified EVs by sandwich ELISA according to the method depicted in Fig. 4a. Specifically, serum containing increasing doses of C4-2B EVs was incubated with Tim4-bound magnetic beads and then EVs were released from Tim4 by adding EDTA. The purified EVs were transferred to a well of 96-well plate where they were recaptured by immobilized Tim4, followed by detection of PSMA on EVs. For comparison with the PSMA-EV measurement without prior EV purification, the same amount of serum containing C4-2B EVs used for EV purification was directly applied to a well coated with Tim4.

Although the sensitivity was slightly reduced, the background signal was decreased when purified EVs were used as a sample instead of serum (Fig. 4b). More importantly, the standard deviation of the background signal was greater when serum was used as a sample compared with when purified EVs were used. Indeed, the background signals varied among 5 donors when PSMA-EVs were measured directly from serum (Supplementary Fig. S4). In contrast, the variation of the background signal was only minimal when the PSMA-EV levels were determined using purified EVs. The differences in the background level among donors observed when PSMA-EVs in serum were directly measured are likely to be caused by the matrix components and ALP activity present in serum, both of which may be variable among individuals. It is also noteworthy that the background and dose-dependent increase of the PSMA-EV signal obtained by measuring purified EVs isolated from the C4-2B EV sample alone were almost the same as those from serum of 5 donors containing C4-2B EVs, addressing the effectiveness of eliminating contaminants in serum by Tim4. Taken all together, we developed a novel sandwich ELISA for detection of PSMA-EVs using EVs purified from serum by Tim4 as a sample, which will allow us to measure their amounts accurately.

### PSMA-EVs in serum of mouse xenograft model for PC

**Figure 5:**
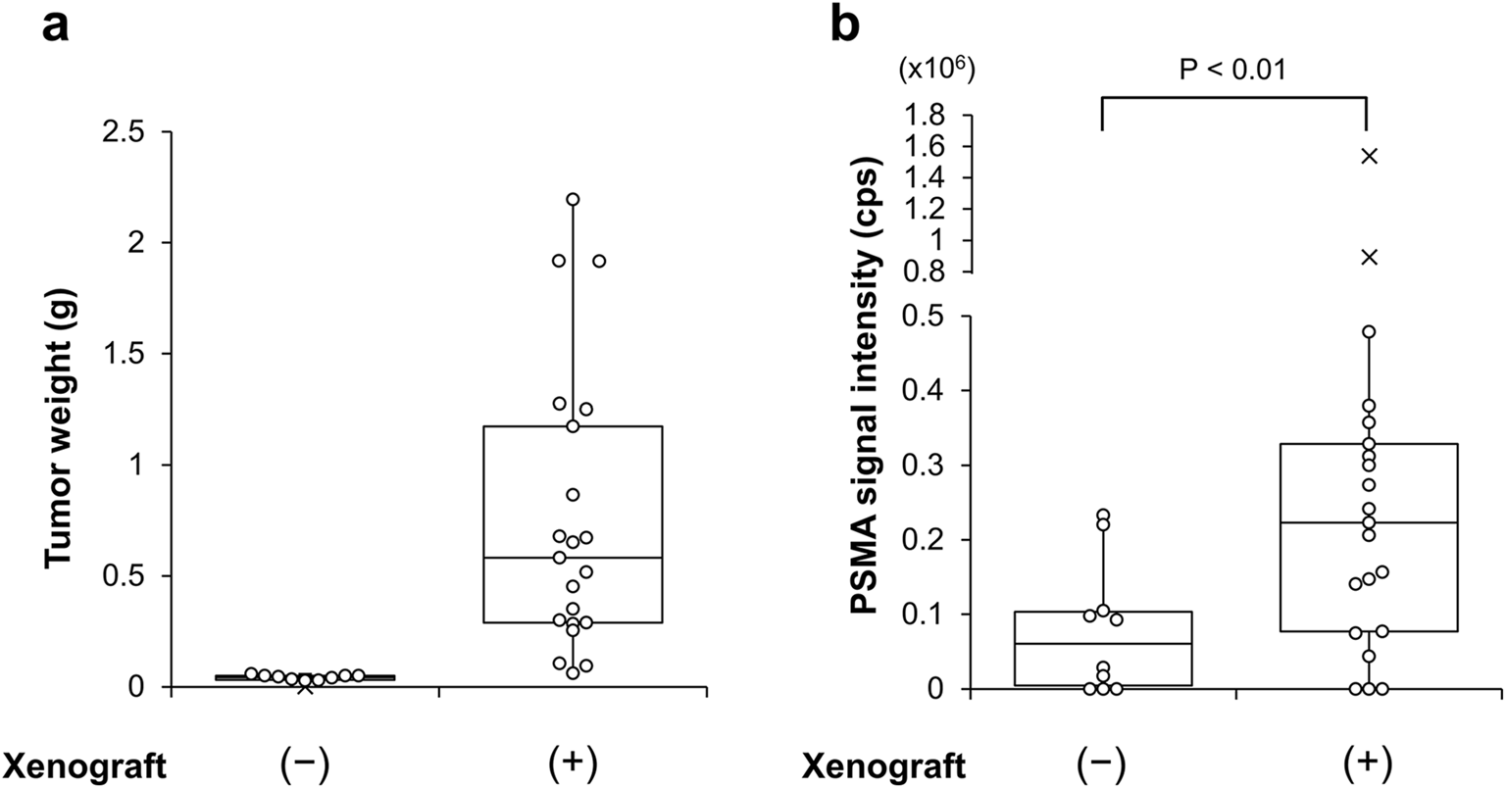
Measurement of the PSMA-EV levels in serum of mouse xenograft model for PC. The C4-2B PC cells were injected into the subcutaneous space of athymic nude mice. After the formation of tumor, blood was collected and the xenograft tissues were removed from control (n = 10) and xenograft mice (n = 21). (**a**) The tumor weight was measured. (**b**) The PSMA-EV levels in serum was determined after purification of EVs from serum by Tim4. Data are shown in the 'box and whiskers' graph with interquartile range (IQR: box), median (thick vertical line in the box) and 1.5 IQR (whiskers). x denotes outliers.

In order to assess the validity of the PSMA-EV sandwich ELISA, we measured the PSMA-EV level in serum of mouse xenograft model for PC. The human PC C4-2B cells were injected into the subcutaneous space of athymic nude mice. After the formation of tumor (Fig. 5a), blood was collected. Measurement of the PSMA-EVs in serum after purification of EVs by Tim4 revealed that the signal intensity was significantly higher in xenograft mice than in control mice (Fig. 5b). These results indicate that PSMA-EVs released from implanted C4-2B cells exist in serum of xenograft mice and that the sandwich ELISA we developed is valid and feasible for measuring human PSMA-EVs.

### PSMA-EVs in serum of PC patients

**Figure 6:**
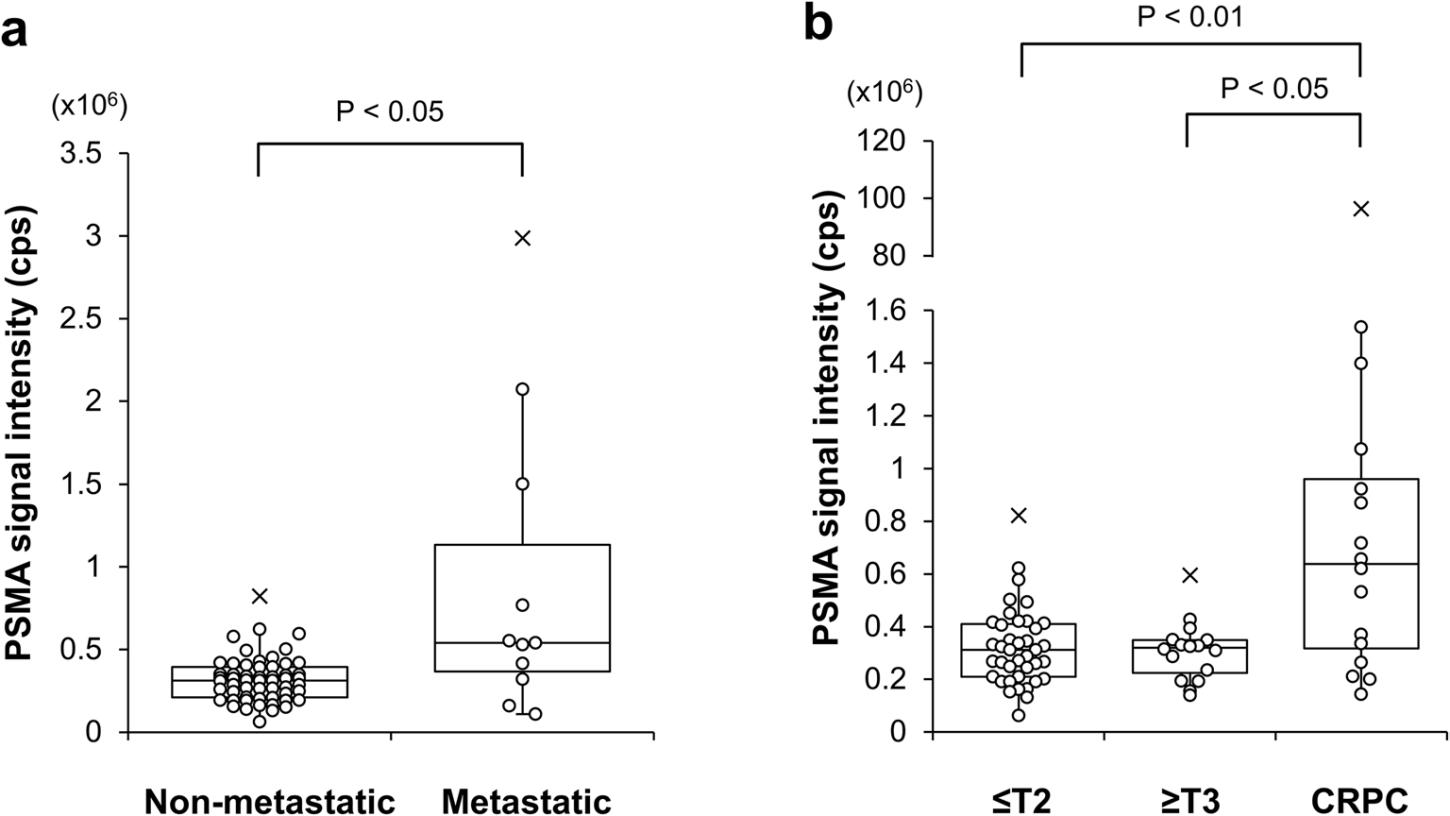
Measurement of the PSMA-EV levels in serum of PC patients. The PSMA-EV levels in serum was determined after purification of EVs from serum by Tim4. Patients with therapy-naïve PC and CRPC were analyzed. Comparisons were made between non-metastatic (n = 55) and metastatic patients (n = 11) (**a**) and among organ-confined PC patients (≤ T2, n = 39), locally-advanced PC patients without metastasis (≥ T3, n = 16) and CRPC patients (n = 16) (**b**). Data are shown in the 'box and whiskers' graph with interquartile range (IQR: box), median (thick vertical line in the box) and 1.5 IQR (whiskers). x denotes outliers.

Using the newly-developed sandwich ELISA, we determined the level of PSMA-EVs in purified EVs isolated from serum of 71 PC patients including 55 therapy-naïve patients and 16 CRPC patients. At initial diagnosis, PSMA-EVs were elevated in therapy-naïve PC patients with metastasis compared with those without metastasis (Fig. 6a). Furthermore, the PSMA-EV levels were significantly higher in CRPC patients than in organ-confined PC patients (pathological T stage less than or equal to T2) and locally-advanced PC patients without metastasis (T stage greater than or equal to T3) (Fig. 6b). These results suggest that the PSMA-EV sandwich ELISA may be helpful to diagnose and monitor metastatic PC and CRPC patients.

### PSMA-EVs in serum of RCC patients

**Figure 7:**
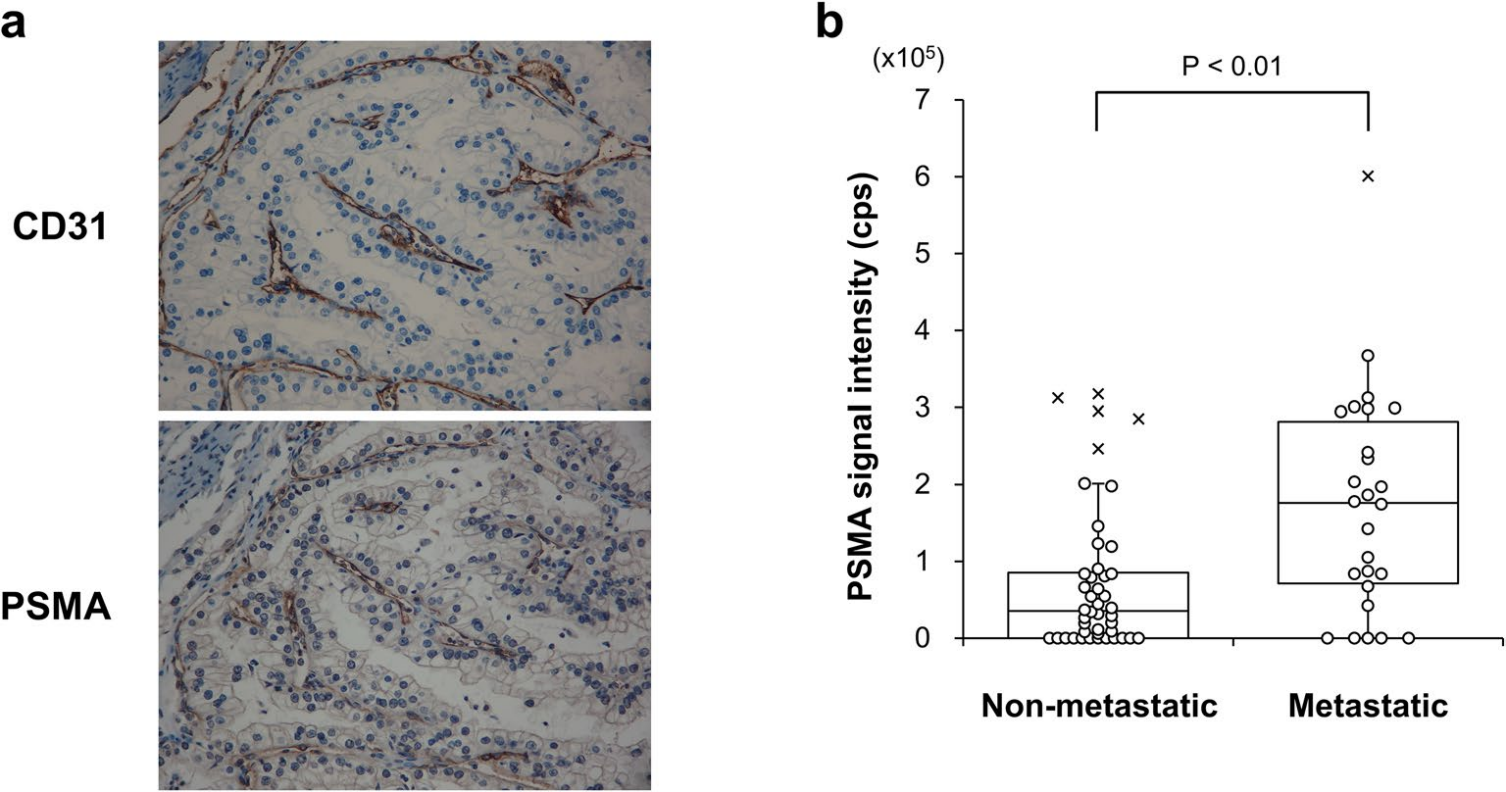
Measurement of the PSMA-EV levels in serum of RCC patients. (**a**) A representative image of CD31 and PSMA immunohistochemical staining is shown. (**b**) The PSMA-EV levels in serum was determined after purification of EVs from serum by Tim4. RCC patients with (n = 26) and without (n = 44) metastasis were analyzed. Data are shown in the 'box and whiskers' graph with interquartile range (IQR: box), median (thick vertical line in the box) and 1.5 IQR (whiskers). x denotes outliers.

PSMA is expressed in the endothelial cells of the neovasculature of RCC^19^ and PSMA-PET/CT has been suggested to be useful to identify the metastatic region of RCC^30^. In order to confirm the PSMA expression in the neovasculature of RCC, we performed immunohistochemical analysis of surgically-resected primary tumors. Although substantial portions of vascular endothelial cells expressing blood vessel marker CD31 were positive for PSMA, those negative for PSMA were also found (Fig. 7a). In addition, there were patients with no PSMA staining in the neovasculature. These results indicate that PSMA expression in the endothelial cells may be variable among patients. As anticipated, no correlation was found between the score of PSMA expression and the stage or grade of RCC (data not shown). However, it was revealed that the PSMA-EV levels at initial diagnosis were higher in RCC patients with metastasis than in those without metastasis (Fig. 7b). These results suggest that PSMA-EVs in serum may reflect the development of the neovasculature in primary and metastatic lesions and that the PSMA-EV sandwich ELISA could be used to diagnose and monitor metastatic RCC patients.

### The PSMA/CD9 ratio in serum of PC and RCC patients

We demonstrated that the PSMA-EV sandwich ELISA we developed is useful to diagnose and monitor metastatic PC and CRPC patients as well as metastatic RCC patients. Unexpectedly, we found that the levels of CD9-positive EVs in purified EVs isolated from the same amount of serum by Tim4 were significantly reduced in metastatic PC and CRPC patients compared with therapy-naïve PC patients, whereas there was no statistical difference in the CD9-EV levels between RCC patients with and without metastasis (Supplementary Fig. S5). As anticipated, the difference between therapy-naïve PC patients and metastatic PC and CRPC patients became more evident, when the PSMA signal was divided by the CD9 signal (PSMA/CD9) (Supplementary Fig. S6). Although the reason for the decrease in CD9-positive EVs in purified EVs remains to be elucidated, the PSMA/CD9 value rather than the PSMA signal intensity might be more useful for differential diagnosis in clinical practice.

## Discussion

PSMA is predominantly expressed in the prostate gland and its expression has been shown to be elevated in PC and correlated with various clinicopathological features of PC patients^13,16,31^.We isolated PSMA-EVs from serum by immunocapture using anti-PSMA antibody and detected them on Western blot using anti-CD9 antibody, demonstrating their presence in serum^25^. Although the number of patients studied was small, we also showed that the PSMA-EV level in serum was higher in advanced PC patients including CRPC patients compared with hormone-naïve PC patients. In order to confirm these findings, we attempted to develop a sandwich ELISA for detection of PSMA-EVs by which measurement could be performed for many samples using a small amount of serum.

Initially, we intended to detect PSMA on EVs captured from serum by antibody recognizing a common EV marker CD9 or CD63. It was indeed easy to develop antibody-based sandwich ELISA capable of measuring PSMA-EVs in serum-free C4-2B EV samples. However, when serum containing C4-2B EVs were applied, the presence of serum in samples even at 10% almost completely inhibited the dose-dependent signals obtained with serum-free samples. This problem so called “matrix effect” is caused by the presence of serum or plasma in the reaction^7^ and needs to be solved to obtain accurate and reproducible results by any immunoassay^32^.

Upon construction of ELISA, the matrix effect usually does not matter, if the target is abundant in samples. Judging from the difficulty in developing antibody-based sandwich ELISA, we speculated that the amount of PSMA-EVs in blood may be much less than expected. We therefore employed Tim4 as a capture molecule to increase the yield of EVs from serum. During the development of the PSMA-EV sandwich ELISA using Tim4, we noticed that the matrix effect was still observed, but was much less than that experienced with the conventional antibody-based method. Since the sensitivity was acceptable even at the highest concentration tested, we decided to measure PSMA-EVs in the presence of 50% serum to increase their yield, which, however, slightly elevated the background signal.

Specificity is a key feature of any immunoassay including sandwich ELISA. The competitive assay demonstrated that the dose-dependent signal is specific to PSMA-EVs and that the background signal may be due to the matrix effect of serum or ALP activity present in serum. In order to further reduce the background by eliminating these contaminants, we determined the PSMA-EV levels on purified EVs released from Tim4 that had captured EVs in serum. As a result, we succeeded in accurately measuring the PSMA-EV levels in serum.

Our results indicate that Tim4-based method is superior to conventional antibody-based methods in terms of higher EV capture efficiency and yield of purified EVs from serum and less matrix effect on measurement. As far as we know, Tim4 seems the best tool to capture highly-purified EVs with minimal contaminants from serum with ease in large quantities. In the present study, we for the first time demonstrated that the use of Tim4-purified EVs as a sample may minimize the background in sandwich ELISA and provide accurate measurement of target EVs, which would be important especially dealing with those with low abundance such as cancer-derived EVs.

The PSMA-EV levels in serum of mouse xenograft model implanted with human PC C4-2B cells expressing PSMA was lower than we expected, based on the tumor per body weight ratio. The EVs released from the tissue into culture medium are referred to as “tissue-exudative extracellular vesicles (Te-EVs)”^33^. Surprisingly, the PSMA levels in the conditioned medium of the xenograft tumors were much higher than those in serum (data not shown), suggesting that PSMA-EVs may not be readily released from xenograft tumors, partly because they are covered with the fibrous capsule and have relatively poor vascular supply.

Using the newly-developed sandwich ELISA, we determined the level of PSMA-EVs in purified EVs isolated from serum in patients with PC. PSMA-EVs were elevated in therapy-naïve PC patients with metastasis compared with those without metastasis, and were significantly higher in CRPC patients than in organ-confined PC patients (≤ T2) and locally-advanced PC patients (≥ T3), which was consistent with the results of our earlier study^25^. We also found that the PSMA-EVs signals were higher in localized PC patients with Gleason score (GS) ≥ 8 than in those with GS ≤ 7 (data not shown). However, there was no statistical difference between BPH and therapy-naïve PC patients (data not shown).

There have been a few reports which developed PSMA-EV detection system and measured their levels in patients with PC. In a report, the PSMA-EV levels were different among BPH and low-, intermediate- and high-risk PC groups^26^. In another report, the PSMA-EV signals were higher in PC patients with GS ≥ 8 than in those with lower Gleason score and were also higher in metastatic PC and CRPC patients^27^. Taken together, despite the difference in the methods and target EVs, we and others reported partly similar findings about the clinical significance of measuring PSMA-EVs in PC patients.

On the other hand, it has been demonstrated that the endothelial cells in the neovasculature of solid tumors including RCC express PSMA^19–21^. We hypothesized that PSMA-EVs secreted from endothelial cells in the neovasculature of tumor could be a biomarker for RCC. We found that the PSMA-EV levels were higher in RCC patients with metastasis than in those without metastasis. Although immunohistochemical analysis revealed that PSMA expression in the endothelial cells of the neovasculature may vary depending on patients, our results suggest that the PSMA-EV measurement would be useful for diagnosis and monitoring of RCC patients and possibly for evaluation of anti-angiogenic agents such as tyrosine-kinase inhibitors. To the best of our knowledge, this is the first report showing that PSMA-EVs can be a marker for the development of the neovasculature in tumors.

PSMA-PET/CT has been the focus of intense research^22^ and is currently used in clinical practice for diagnosis and monitoring of PC patients such as metastatic PC and CRPC patients^34^. The potential use of PSMA-PET/CT for RCC patients has been also proposed^30^. Importantly, the findings in the present study that PSMA-EVs in serum were elevated in metastatic PC and CRPC patients and in metastatic RCC patients are consistent with the proposed use of PSMA-PET/CT. Once the correlation of the data between PSMA-PET/CT and PSMA-EV is established by large-scale prospective clinical studies, PSMA-EV testing could be an indication marker for PET/CT that is expensive for repeated use.

In conclusion, we developed a sandwich ELISA for detection of PSMA-EVs with low background and with proven specificity, which will allow us to accurately determine their levels in serum. Our results suggest that the use of highly-purified EVs isolated from serum by using Tim4 as a sample would help reduce the background and matrix effect when measuring EVs existing in trace amount in blood. Using the newly-developed PSMA-EV sandwich ELISA, we demonstrated that the PSMA-EV levels were higher in metastatic PC and CRPC patients as well as in metastatic RCC patients, suggesting their usefulness for diagnosis and monitoring of PC and RCC. Large-scale prospective clinical studies will further confirm the validity of the PSMA-EV sandwich ELISA in the management of PC patients and RCC and other solid tumor patients.

## Methods

All experimental protocols were approved by the Institutional Review Board of Gifu University and Tokyo Metropolitan Institute of Gerontology. All methods were carried out in accordance with relevant guidelines and regulations.

### Patients and normal controls

This study was approved by the Bioethics Committees of Gifu University (29-410) and Tokyo Metropolitan Institute of Gerontology (6177) and a written informed consent was obtained from all patients. All methods were carried out in accordance with relevant guidelines and regulations (Declaration of Helsinki). Ninety patients suspicious of PC due to either abnormal MRI findings or elevated PSA levels were recruited. Blood was collected from patients prior to biopsy. After biopsy, 66 patients and 24 patients were pathologically diagnosed as localized PC and benign prostatic hyperplasia (BPH), respectively. In addition, 16 patients of CRPC were recruited and blood was taken after diagnosis. Seventy patients with RCC consisting of those with (n = 26) and without (n = 44) metastasis were recruited and blood was collected. Serum was separated from whole blood by centrifugation at 1800 × *g* for 20 min and subsequently centrifuged at 16,500 × *g* for 30 min to remove cell debris and large EVs. Samples were stored at – 80 °C until use. As normal control samples, serum of 6 healthy donors were purchased from KAC (Kyoto, Japan, CTSER018).

### Tim4 and antibodies

Mouse TIM4/Human Fc Chimera, recombinant (Tim4) was obtained from FUJIFILM Wako Pure Chemical Corporation (Osaka, Japan, 137-18511). Anti-PSMA antibodies were purchased from Abcam (Cambridge, UK, ab66912, clone GCP-05), BioLegend (CA, USA, 342502, clone LNI-17), MBL (Nagoya, Japan, K0142-3, clone 107-14), Merck Millipore (MA, USA, MABC291, clone 3/A12), Miltenyi Biotec (Bergisch Gladbach, Germany, custom-ordered, clone REA408), R&D Systems (MN, USA, AF4234, sheep-polyclonal). Anti-CD9 antibodies were from BioLegend (312102, clone HI9a) and MBL (MEX001-3, clone A100-4). Anti-CD63 antibody was purchased from MBL (MEX002-3, clone C047-1). Alkaline phosphatase (ALP)-labeled anti-CD9 and -CD63 antibodies were from MBL (MEX001-12, clone A100-4 and MEX002-12, clone C047-1, respectively).

### Tim4 and antibody labeling

Tim4 was labeled with biotin using Biotin labeling kit-SH (Dojindo Laboratories, Kumamoto, Japan, LK10). Anti-PSMA antibodies were labeled with ALP using Alkaline phosphatase labeling kit-SH (Dojindo Laboratories, LK13).

### Cell culture and EV isolation

Human androgen-refractory PC cell line C4-2B was obtained from the MD Anderson Cancer Center (Houston, TX, USA). Cell culture condition and EV isolation method from conditioned medium were described previously^35^. Briefly, C4-2B cells were cultured in RPMI1640 medium containing 10% of fetal bovine serum and penicillin/streptomycin. Prior to EV isolation, cells were cultured in advanced RPMI1640 medium (Thermo Fisher Scientific, MA, USA, 12633012) without serum. The conditioned medium was subjected to filtration through 0.22 µm membrane, followed by sequential centrifugations. EVs pelleted by the final ultracentrifugation at 100,000 × *g* for 70 min were washed twice, resuspended in PBS (−) and stored at – 80 °C until use.

### Plate-based sandwich ELISA (capture molecule/detection antibody: Tim4/CD9 or CD63, CD9/CD9 or CD63, Tim4/PSMA, CD9/PSMA)

Each well of the white MaxiSorp ELISA plate (Thermo Fisher Scientific, 436110) was coated with 125 ng of Tim4 recombinant protein or anti-CD9 antibody (HI9a or A100-4) in 100 µL of carbonate buffer (pH 9.6) overnight at 4 °C. After washing 3 times with TBST (20 mM Tris–HCl, pH 7.4, 150 mM NaCl, 0.01% Tween20, 400 µL), each well was incubated with 1% BSA/TBS (100 µL) for 1 h with shaking at room temperature. After washing with TBST (400 µL), serum, C4-2B EVs, serum containing C4-2B EVs or purified EVs prepared by antibody-bound beads (50 µL) was added to a well containing 2% BSA/TBS supplemented with 4 mM CaCl_2_ (50 µL). On the other hand, the purified EVs prepared by Tim4-bound beads (50 µL) were added to a well containing 2% BSA/TBS supplemented with 40 mM CaCl_2_ (50 µL) in order to neutralize EDTA in samples. After shaking the plate for 1 h at room temperature, each well was washed 5 times with TBST supplemented with 2 mM CaCl_2_ (400 µL). Then, ALP-labeled anti-CD9 or -CD63 antibody (MBL) diluted in 1% BSA/TBS supplemented with 2 mM CaCl_2_ (100 µL, dilution 1:2000) or ALP-labeled anti-PSMA antibody diluted in 1% BSA/TBS supplemented with 2 mM CaCl_2_ (100 µL, dilution 1:1000) was added into well, followed by incubation of the plate for 1 h with shaking at room temperature. After washing 5 times with TBST supplemented with 2 mM CaCl_2_ (400 µL), 50 µL of CDP-Star substrate with Emerald II enhancer (Thermo Fisher Scientific, T2216) was added into well and chemiluminescence was recorded by the EnVision multilabel reader (PerkinElmer, MA, USA).

### Bead-based EV purification by Tim4 and anti-CD9 antibody

To isolate EVs from serum by Tim4 or anti-CD9 antibody, 125 ng of biotinylated Tim4 or biotinylated anti-CD9 antibody (HI9a or A100-4) was conjugated to 75 µg of Dynabeads MyOne streptavidin C1 magnetic beads (Thermo Fisher Scientific, 65001). The beads were resuspended in 50 µL of 2% BSA/TBS supplemented with 4 mM CaCl_2_ and combined with 50 µL of serum or serum containing increasing doses of C4-2B EVs in a well of 96-well polypropylene microplate (Greiner Bio-One International GmbH, Kremsmunster, Austria, 651201), followed by shaking for 1 h at room temperature. The Tim4-bound beads were washed 5 times with 200 µL of TBST supplemented with 2 mM CaCl_2_ using DynaMag-96 side skirted magnet (Thermo Fisher Scientific, 12027). Then, bound EVs were eluted with 50 µL of EDTA elution buffer (20 mM Tris–HCl, pH 7.4, 150 mM NaCl, 2 mM EDTA). On the other hand, the antibody-bound beads were washed 5 times with 200 µL of TBST using the magnet plate and bound EVs were eluted with 46.5 µL of 0.1 M glycine–HCl solution (pH 2.5–3.0), followed by neutralization with 3.5 µL of 1 M Tris–HCl (pH 8.0) buffer.

### Bead-based sandwich ELISA for detection of CD9 and CD63 on purified EVs

The CD9 and CD63 levels on purified EVs were determined by bead-based sandwich ELISA. Briefly, 50 ng of biotin-labelled CD9 or CD63 antibody (MBL) was conjugated to streptavidin magnetic beads (5 µL) using ExoCap streptavidin kit (MBL, MEX-SA) and the beads were resuspended in treatment buffer included in the kit (50 µL). Two or 10 µL of purified EVs diluted to 50 µL with TBS were incubated with magnetic beads conjugated with anti-CD9 or -CD63 antibody (MBL), respectively, in the well of white Non-binding surface microplate (Corning, NY, USA, 3990) for 1 h at room temperature. After washing with TBST (200 µL), captured EVs were reacted with ALP-conjugated anti-CD9 or -CD63 antibody (MBL) in 1% BSA/TBS (100 µL). After washing with TBST (200 µL), 50 µL of CDP-Star substrate with Emerald II enhancer was added into the well and chemiluminescence was determined using Envision microplate reader (Perkin Elmer).

### Mouse xenograft model

This study was approved by the Animal Experiment Committee of the Tokyo Metropolitan Institute of Gerontology and carried out in compliance with the ARRIVE guidelines. Male BALB/c-nu mice aged 4 weeks were purchased from Charles River Laboratories Japan (Yokohama, Japan). C4-2B cells (1 × 10^6^) mixed with Matrigel (BD Biosciences, NJ, USA, 354234) were subcutaneously injected into the right flank of mice. Tumor growth was monitored twice per week with caliper. Two months after injection of cells, mice were sacrificed. Blood was collected and the xenograft tissues were taken and weighed.

### Immunohistochemical analysis

This study was approved by the Bioethics Committees of Gifu University (28-149). Formalin-fixed paraffin-embedded surgically resected tissue specimens from RCC patients (n = 20) were immunohistochemically analyzed for PSMA and CD31 expression. After deparaffinization, the tissue sections (4 µm) were processed in the Ventana benchmark ultra automatic stainer (Ventana Medical Systems, AZ, USA) according to the manufacturer’s instruction. Briefly, the antigen retrieval was conducted in Cell conditioning 1 (CC1) buffer (Tris–EDTA, pH 8.5, Ventana) for 60 min at 95 °C. The sections were then incubated with anti-PSMA antibody (Abcam, 1:500 in dilution) or anti-CD31 antibody (Dako, CA, USA, 1:100 in dilution) for 1 h at 25 °C, followed by amplification and visualization using I-View DAB universal kit, which is based on the labeled streptavidin–biotin method (Roche, Basel, Switzerland). The sections were counterstained with Mayer’s hematoxylin. Negative control tissue sections were prepared by omitting the primary antibody. PSMA staining was scored by the intensity (1, 0+; 2, 1+; 3, 2+; 4, 3+) and percentage (1, 0–25%; 2, 26–50%; 3, 51–75%; 4, 76–100%) under 200× magnification. The score of intensity multiplied by that of percentage was used as the score for PSMA expression.

### Statistical analysis

Statistical differences were determined by Kruskal–Wallis test followed by Steel–Dwass multiple comparison test (for comparison among organ-confined and locally-advanced PC and CRPC patients) or Brunner-Munzel test (for comparison between control and xenograft mice and for comparison between non-metastatic and metastatic PC and RCC patients). *P* < 0.05 was considered statistically significant.

## Supplementary Information

Supplementary Information.

## Data Availability

The data that support the findings of this study are available from the corresponding author upon reasonable request.

## Abbreviations

ABs: Apoptotic bodies
BPH: Benign prostatic hyperplasia
CRPC: Castration-resistant prostate cancer
EVs: Extracellular vesicles
EXs: Exosomes
MPs: Microparticles
PC: Prostate cancer
PSMA: Prostate-specific membrane antigen
PSMA-EVs: PSMA-expressing EVs
PSMA-PET/CT: PSMA-positron emission tomography/computed tomography
RCC: Renal cell carcinoma
Tim4: T-cell immunoglobulin domain and mucin domain-containing protein 4
