# Supplementary material for: Diagnostic potential of serum extracellular vesicles expressing prostate-specific membrane antigen in urologic malignancies
Source: Sci Rep. 2021 Jul 22;11:15000. doi: 10.1038/s41598-021-94603-9 (PMC8298409; doi:10.1038/s41598-021-94603-9)
Supplement: Supplementary file 1 — Supplementary Information. [file 41598_2021_94603_MOESM1_ESM.pdf]

# Supplementary Information

# Diagnostic potential of serum extracellular vesicles expressing prostate-specific membrane antigen in urologic malignancies

Kyojiro Kawakami[1], Yasunori Fujita[2], Taku Kato[3], Kengo Horie[4], Takuya Koie[4], Keitaro Umezawa[1], Hiroki Tsumoto[1], Yuri Miura[1], Yasuo Katagiri[5], Tatsuhiko Miyazaki[5], Ikuroh Ohsawa[2], Kosuke Mizutani[4, *], Masafumi Ito[2, *]

[1] Research Team for Mechanism of Aging, Tokyo Metropolitan Institute of Gerontology, 35-2 Sakae-cho, Itabashi-ku, Tokyo 173-0015, Japan
[2] Biological Process of Aging, Tokyo Metropolitan Institute of Gerontology, 35-2 Sakae-cho, Itabashi-ku, Tokyo 173-0015, Japan
[3] Department of Urology, Asahi University Hospital, 3-23 Hashimotocho, Gifu, Gifu 500-8523, Japan
[4] Department of Urology, Gifu University Graduate School of Medicine, 1-1 Yanagido, Gifu, Gifu 501-1193, Japan
[5] Department of Pathology, Gifu University Hospital, 1-1 Yanagido, Gifu, Gifu 501-1193, Japan

* Corresponding author

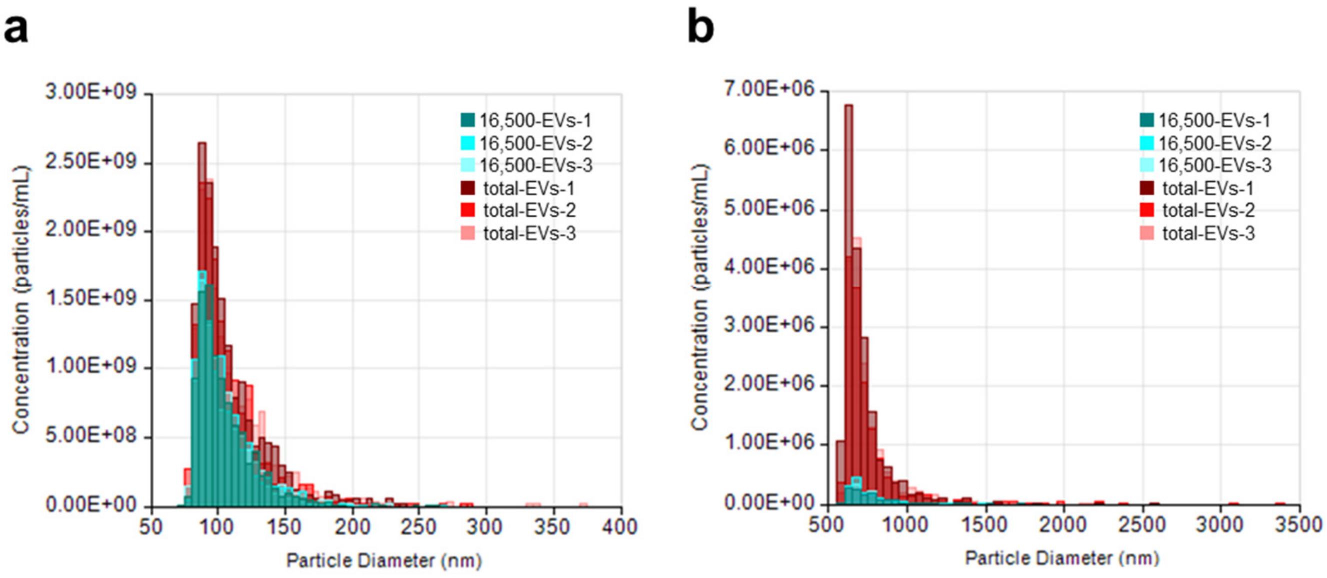


**Supplementary Figure S1. Particle size distribution of EVs isolated from serum with or without prior centrifugation at 16,500 x g.** EVs were isolated from the same pooled serum by ultracentrifugation (100,000 x g, 75 min.) with or without prior centrifugation at 16,500 x g (called 16,500-EVs and total-EVs, respectively). The size distribution of small EVs (**a**) and large EVs (**b**) was measured by qNano nanoparticle analyzer using NP150 and NP1000 nanopore, respectively.

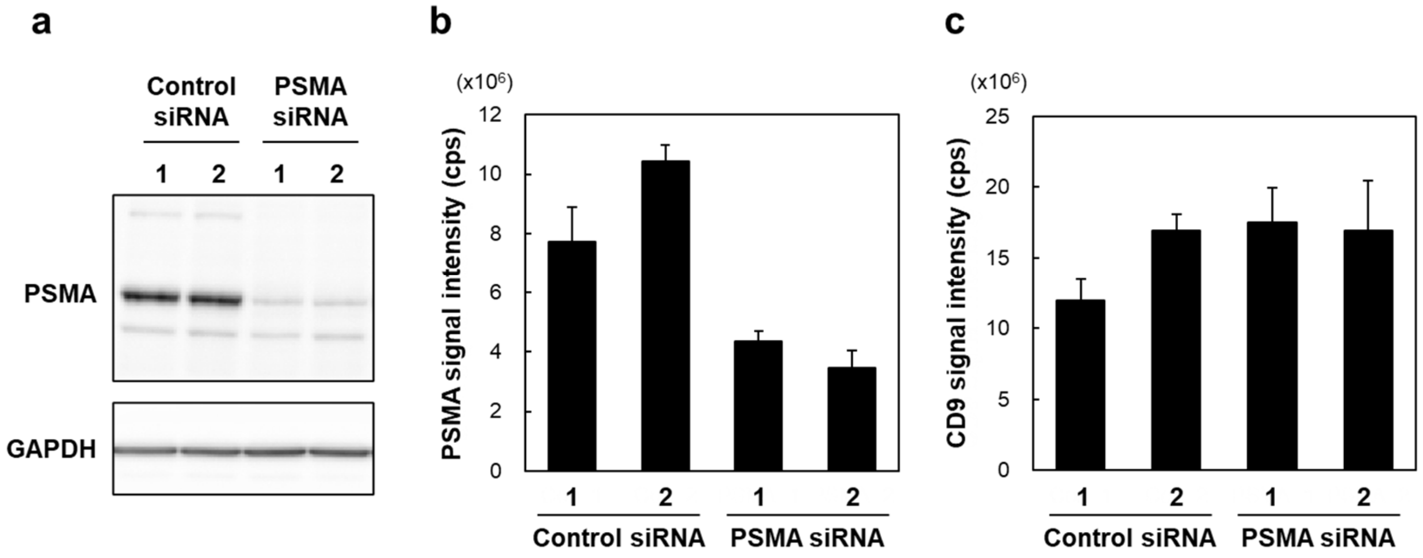


**Supplementary Figure S2. Measurement of the PSMA and CD9 levels on EVs in conditioned medium of PSMA knockdown C4-2B cells.** Western blot analysis of cell lysates from C4-2B cells transfected with control or PSMA siRNAs (**a**). Full-length images of the blots are presented in Supplementary Fig. S7. PSMA (**b**) and CD9 (**c**) levels on EVs in conditioned medium (n=5) were measured by PSMA-EV and CD9-EV sandwich ELISA, respectively.

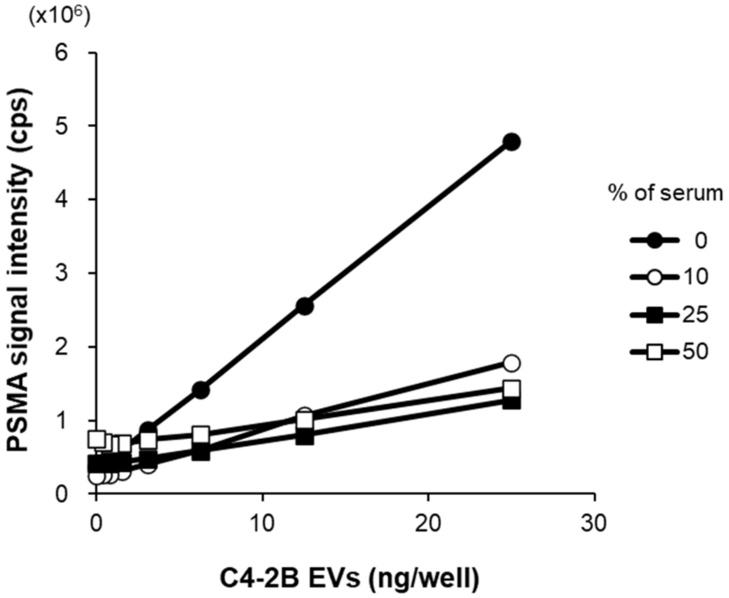


**Supplementary Figure S3. Effect of serum on the anti-CD9 antibody-based PSMA-EV sandwich ELISA.** Increasing doses of C4-2B EVs were applied to a well of 96-well plate coated with anti-CD9 antibody (HI9a). After washing, ALP-labeled anti-PSMA antibody (REA408) was added to the well, followed by detection of chemiluminescence in the presence of ALP substrate. Measurement of PSMA-EVs was performed in the absence and presence of increasing concentration of pooled serum from 6 normal donors (0, 10, 25 and 50%).

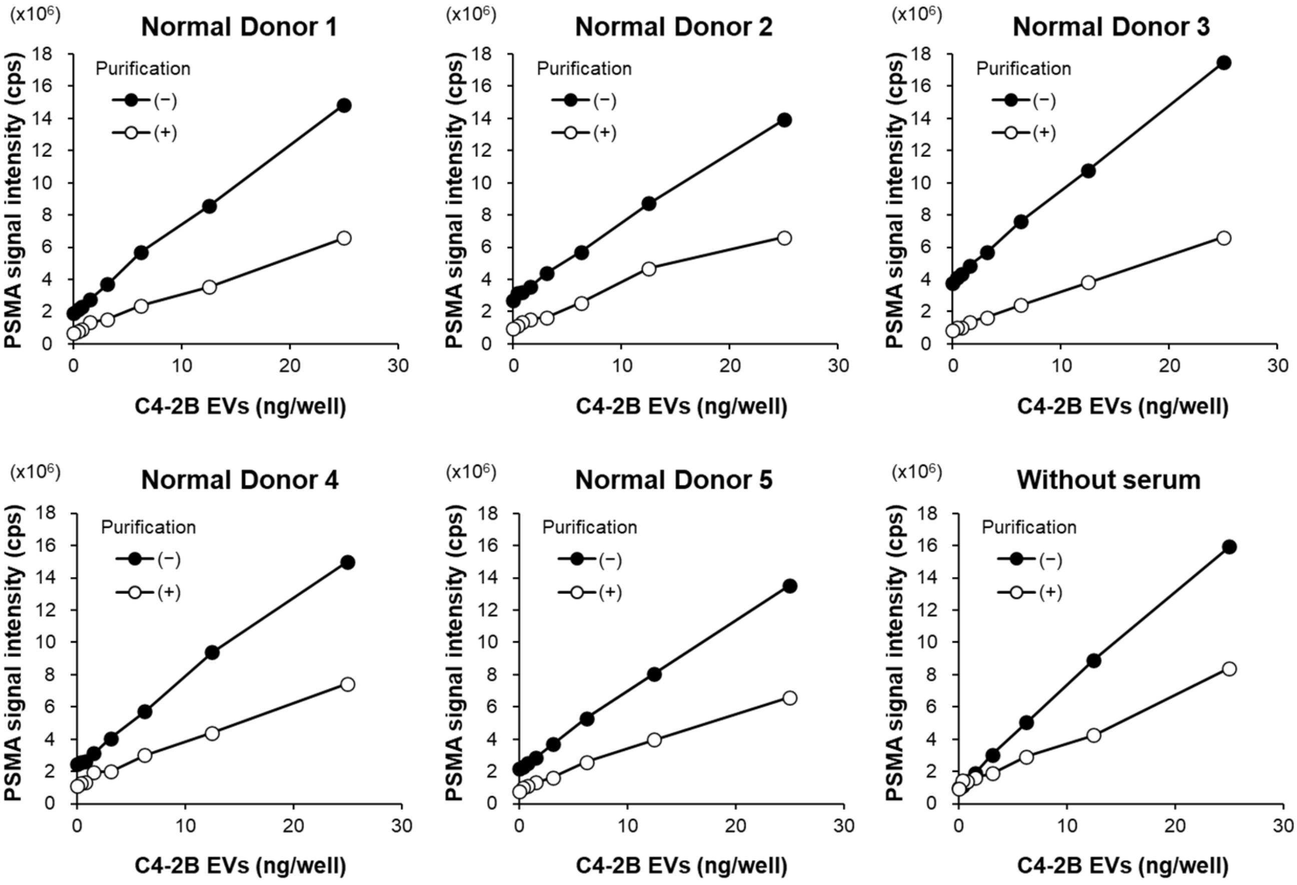


**Supplementary Figure S4. PSMA-EV sandwich ELISA with and without purification of EVs from serum: A comparison among 5 normal donors.** Serum containing increasing doses of C4-2B EVs was prepared for each normal donor (n=5). EVs were purified from the serum containing C4-2B EVs by Tim4. Serum containing increasing doses of C4-2B EVs (50 μL) or EVs purified from the same amount of the serum (50 μL) was subjected to plate-based PSMA-EV sandwich ELISA. Measurement of PSMA-EVs in the C4-2B EV samples and in purified EVs prepared from the same C4-2B EV samples by Tim4 was also performed (Lower most right panel).

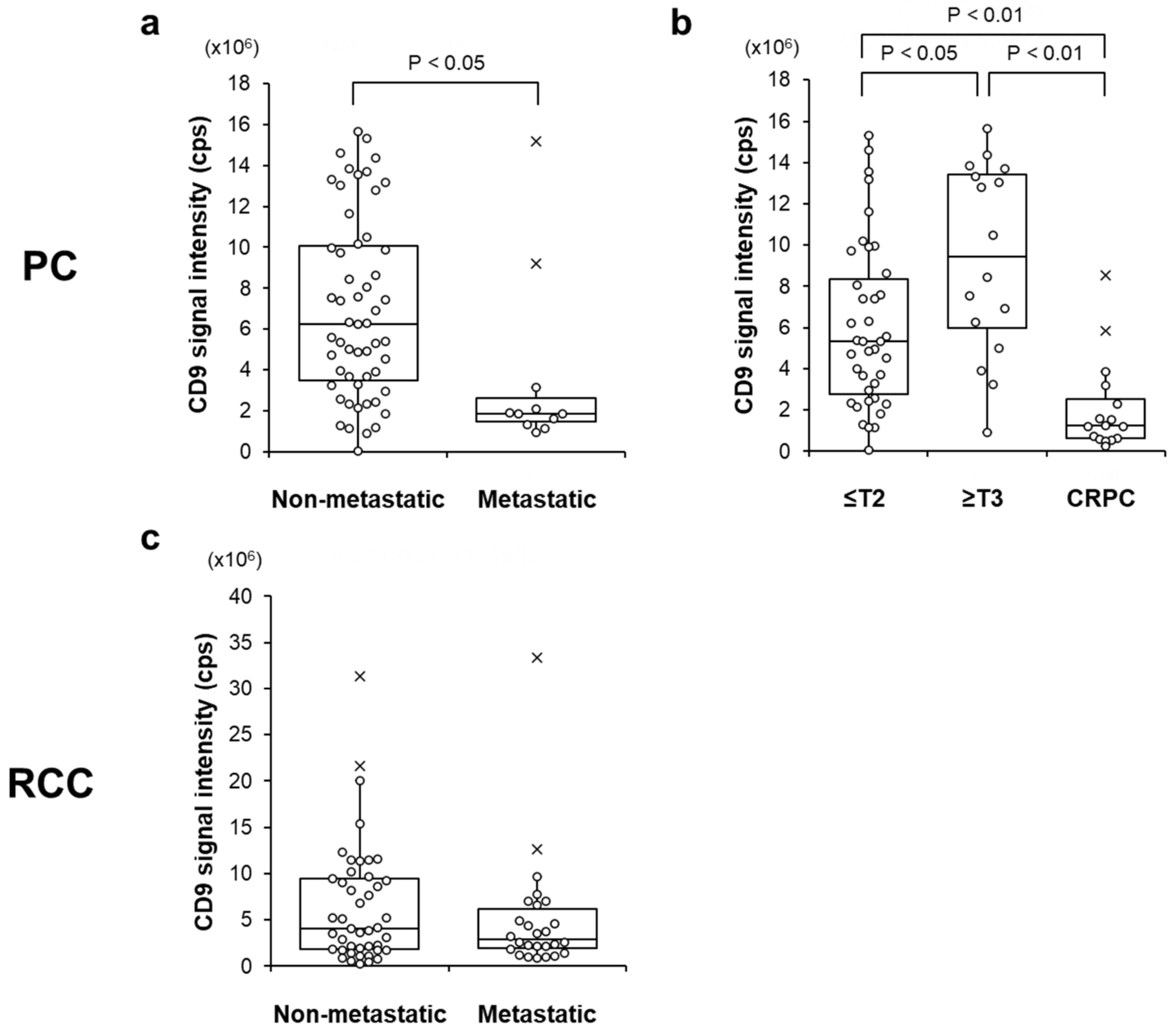


**Supplementary Figure S5. Measurement of the CD9 levels in purified EVs from serum of PC and RCC patients.** The CD9 levels in EVs purified by Tim4 from serum of PC (**a** and **b**) and RCC (**c**) patients were analyzed by bead-based sandwich ELISA. Data are shown in the 'box and whiskers' graph with interquartile range (IQR: box), median (thick vertical line in the box) and 1.5 IQR (whiskers). x denotes outliers.

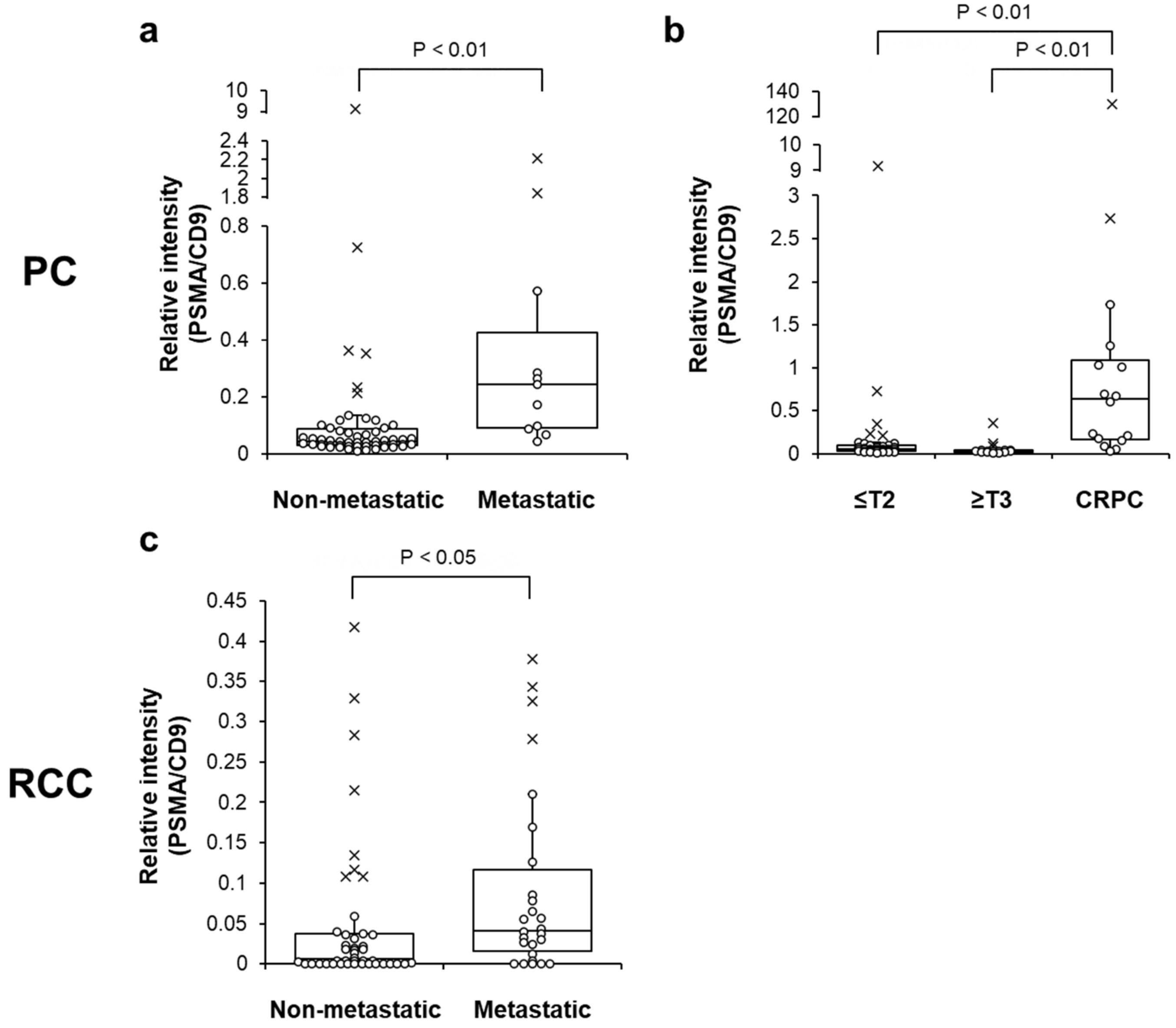


**Supplementary Figure S6. The PSMA/CD9 value in patients with PC and RCC patients.** The PSMA signal intensity shown in Figures 6 and 7 was divided by the CD9 signal intensity shown in Supplementary Figure 3 in patients with PC (**a** and **b**) and RCC (**c**). Data are shown in the 'box and whiskers' graph with interquartile range (IQR: box), median (thick vertical line in the box) and 1.5 IQR (whiskers). x denotes outliers.

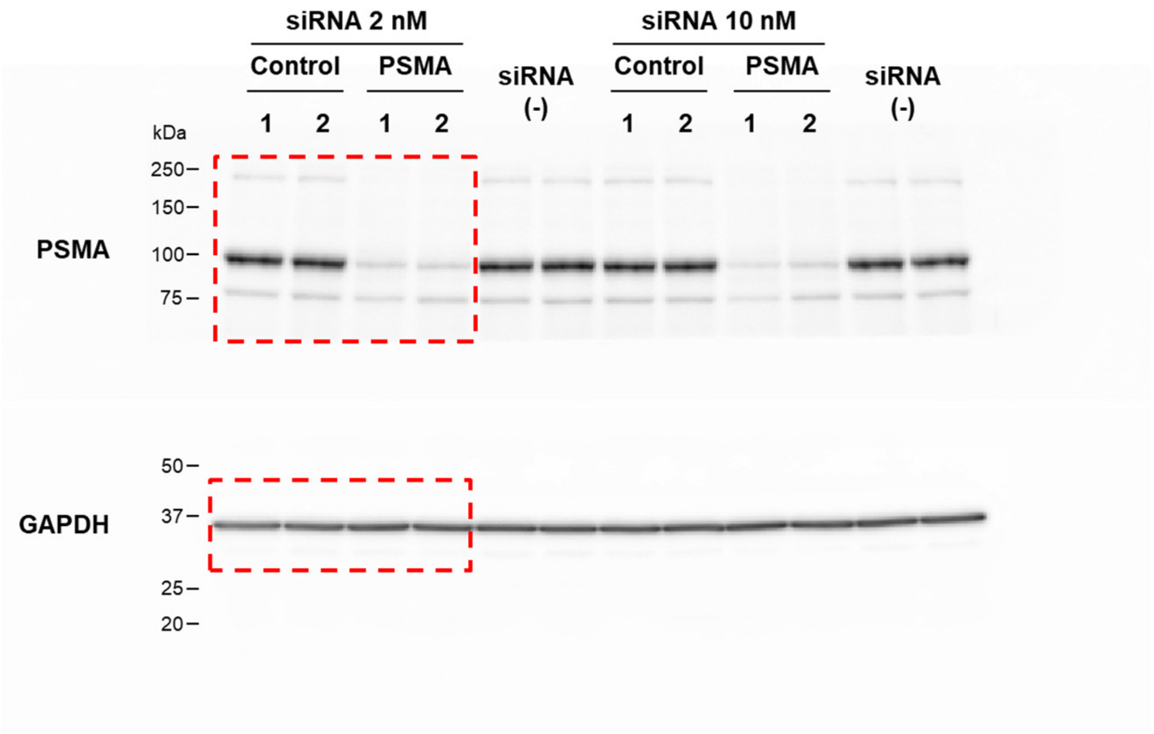


**Supplementary Figure S7. Full-length images of the blots presented in Supplementary Figure S2.** A single gel was transferred to a single membrane. The membrane was cut into two pieces, upper and lower, each of which was then probed with each antibody.

## Supplementary Methods

### Particle size analysis

Particle size distribution analysis of EVs was performed with the qNano nanoparticle analyzer system (IZON Science, Christchurch, New Zealand). After dilution in PBST, the size of EVs was measured using the NP150 (size range: 70-420 nm) and NP1000 (size range: 490-2900 nm) nanopore. The data were analyzed by Izon Control Suite 3.3.2 software according to the manufacturer's instructions.

### Small interfering RNA transfection

Small interfering RNAs (siRNAs) for human PSMA (FOLH1) gene (D-005881-03-0002 and D-005881-04-0002) and non-targeting siRNA control gene (D-001210-01-05 and D-001210-02-05) were purchased from Horizon Discovery (Cambridge, UK). siRNAs were transfected into C4-2B cells at 2 nM using Lipofectamine RNAiMax transfection reagent (Thermo Fisher Scientific, 13778150) according to the manufacturer's instructions. After 6 h incubation, medium was changed to RPMI1640 supplemented with 10% EV- depleted FBS. After 2-day culture, conditioned medium was collected and centrifuged 2,000 x g for 10 min and the supernatant was centrifuged 12,000 x g for 30 min. Then, the supernatant was used for measurement of PSMA-EVs.
